# Supplementary material for: Single-cell image-based genetic screens systematically identify regulators of Ebola virus subcellular infection dynamics
Source: bioRxiv. 2024 Apr 7:2024.04.06.588168. Preprint. [Version 1] doi: 10.1101/2024.04.06.588168 (PMC11014611; doi:10.1101/2024.04.06.588168)
Supplement: 1 [file NIHPP2024.04.06.588168V1-supplement-1.pdf]

bioRxiv preprint doi: https://doi.org/10.1101/2024.04.06.588168; this version posted April 7, 2024. The copyright holder for this preprint (which was not certified by peer review) is the author/funder, who has granted bioRxiv a license to display the preprint in perpetuity. It is made available under aCC-BY 4.0 International license.

Supplementary Information

Table S1. Per-gene mean cumulative delta AUC scores and p-values for VP35 protein, VP35 RNA FISH, and c-Jun channels in genome-wide screen.
Table S2. Per-gene mean cumulative delta AUC scores for all predefined features.
Table S3. Per-gene mean random forest regression results for VP35 RNA FISH and c-Jun predictions.
Table S4. Per-gene mean deep learning predictions of VP35 subcellular protein localization and associated ordinal chi square statistics and p-values.
Table S5. Per-gene mean cumulative delta AUC scores for unsupervised autoencoder features.
Table S6. Per-gene mean cumulative delta AUC scores for supervised transfer learned features.
Table S7. Mean delta AUCs per gene for all features with or without matching to infection level.
Table S8. Per-gene mean delta AUC scores and p-values for VP35 protein and VP35 RNA FISH channels in all secondary screen conditions.
Supplementary Figures 1-4.

bioRxiv preprint doi: https://doi.org/10.1101/2024.04.06.588168; this version posted April 7, 2024. The copyright holder for this preprint (which was not certified by peer review) is the author/funder, who has granted bioRxiv a license to display the preprint in perpetuity. It is made available under aCC-BY 4.0 International license.

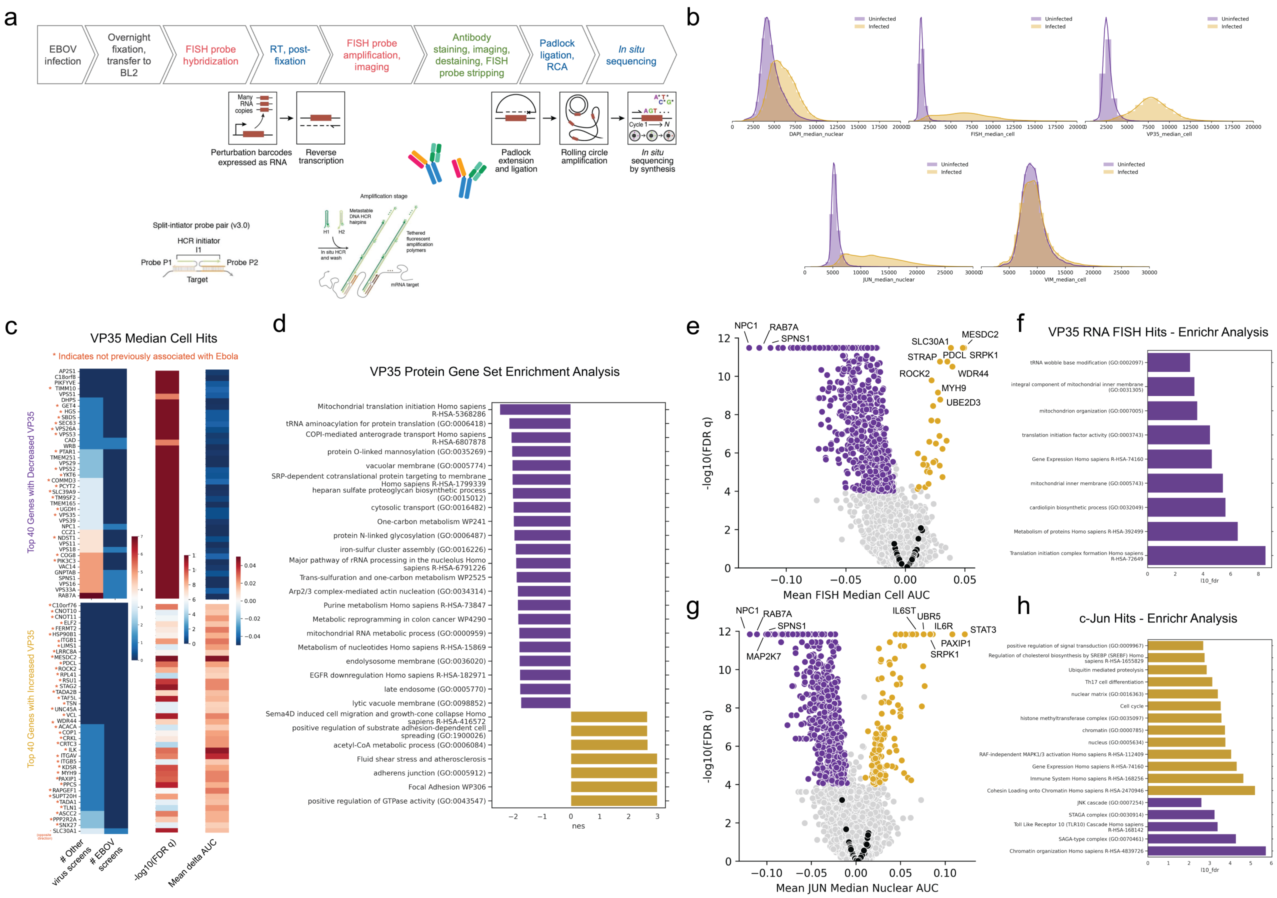

Figure S1. (A) Integration of optical pooled screening workflow with RNA FISH detection using HCR amplification. (B) Histograms of intensity features in five channels for non-targeting controls cells that were infected or not infected in the genome-wide optical pooled screen. (C) Top 40 hits with increased or decreased VP35 protein levels and the number of non-Ebola virus genetic screens or Ebola-specific genetic screens they scored in. Genes not previously associated with Ebola in the literature are marked with an orange asterisk. (D) Gene set enrichment analysis of genes with significantly decreased (purple) or increased (gold) Ebola virus VP35 protein levels. (E) Volcano plot showing genes that scored significantly for changes in VP35 RNA levels by FISH. (F) Enrichr analysis of gene ontology terms significantly enriched in genes that reduced VP35 RNA levels. (G) Volcano plot showing genes that scored significantly for changes in c-Jun levels. (H) Enrichr analysis of gene ontology terms significantly enriched in genes that reduced or enhanced c-Jun levels.

bioRxiv preprint doi: https://doi.org/10.1101/2024.04.06.588168; this version posted April 7, 2024. The copyright holder for this preprint (which was not certified by peer review) is the author/funder, who has granted bioRxiv a license to display the preprint in perpetuity. It is made available under aCC-BY 4.0 International license.

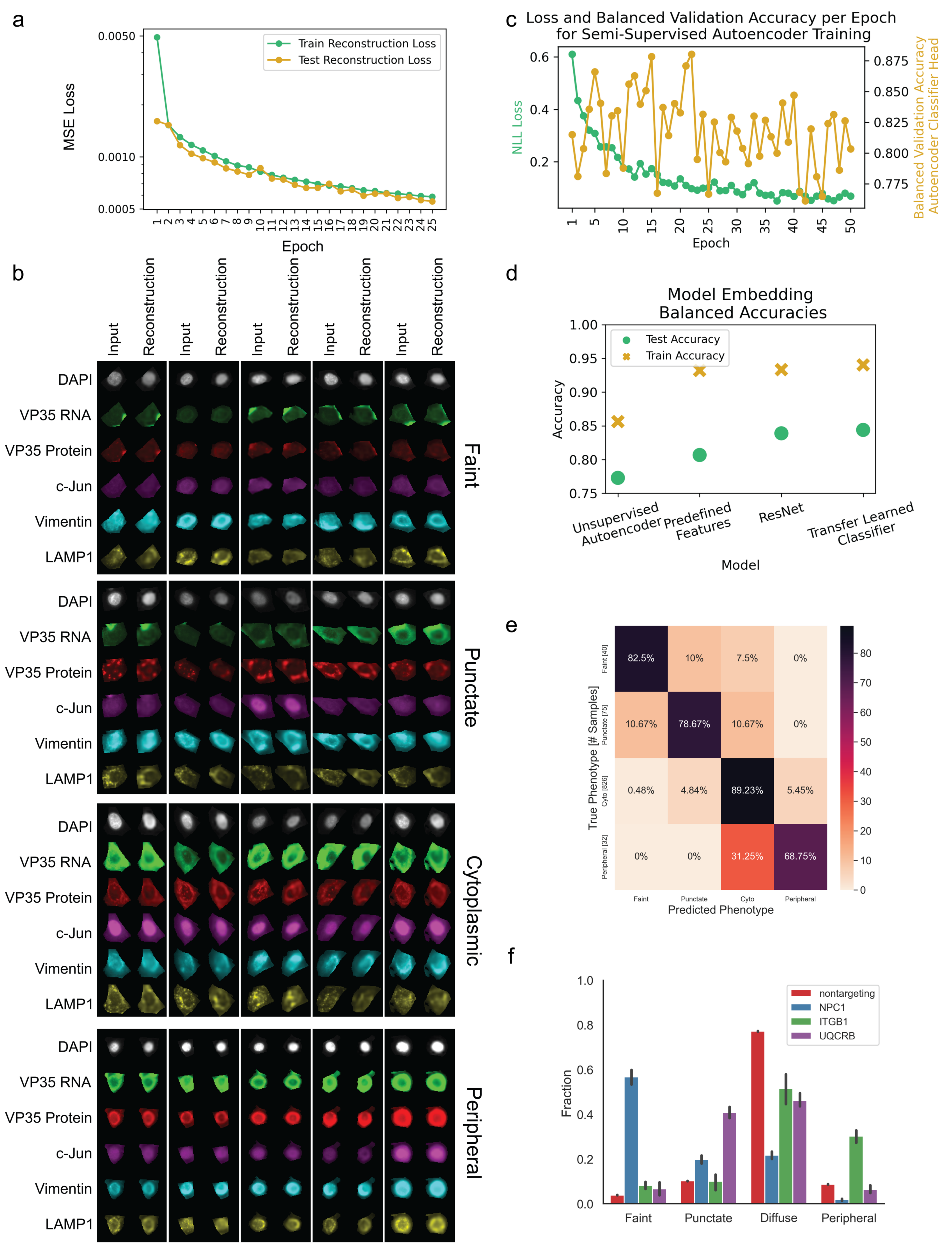

Figure S2. (A) Fully unsupervised autoencoder reconstruction losses for training and test sets across 25 epochs. (B) Examples of manually labeled faint, punctate, cytoplasmic, and peripheral input cell images with accompanying unsupervised autoencoder reconstructions. (C) Fine-tuned autoencoder trained using negative log likelihood loss with balanced validation accuracy also reported across 50 epochs of training. (D) Best model train and test set accuracies for the VP35 protein localization prediction task using SVMs on latent embeddings from the unsupervised autoencoder, predefined features, a Resnet-50 architecture trained on the prediction task, or the fine-tuned autoencoder. Predefined features include intensity, correlation, and texture morphological features similar to those previously described for Cell Painting (Bray et al., 2016). (E) Confusion matrix of model predictions vs manually labeled classifications on model test set. (F) Proportion of cells in each VP35 localization category for non-targeting controls and the genes with the largest proportion of faint (NPC1), punctate (UQCRB), and peripheral (ITGB1) cells. Error bars indicate SEM across sgRNAs targeting the same gene.

bioRxiv preprint doi: https://doi.org/10.1101/2024.04.06.588168; this version posted April 7, 2024. The copyright holder for this preprint (which was not certified by peer review) is the author/funder, who has granted bioRxiv a license to display the preprint in perpetuity. It is made available under aCC-BY 4.0 International license.

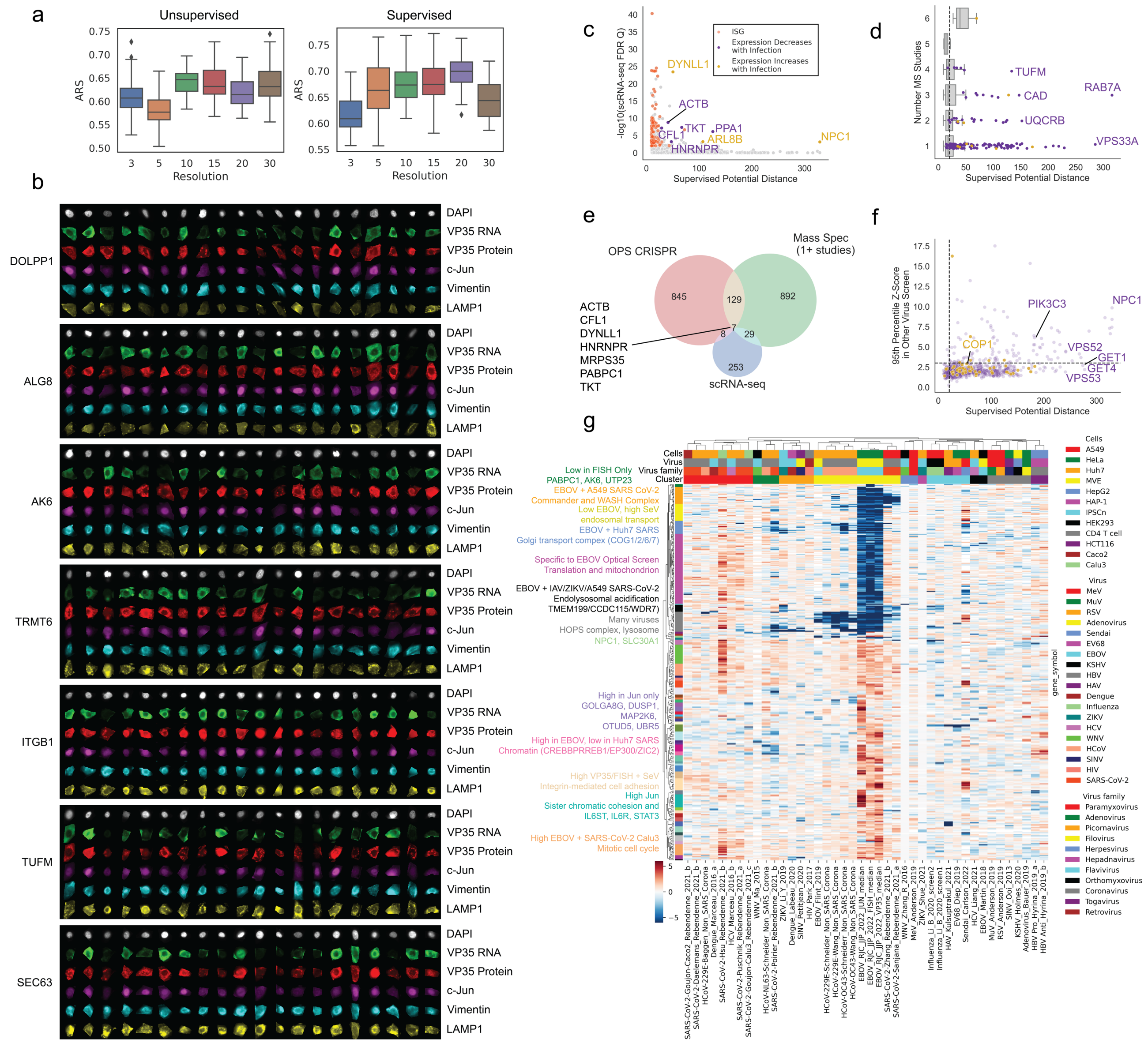

Figure S3. (A) Adjusted Rand score for Leiden clustering at different resolutions. (B) Additional single-cell images of select genetic knockouts from the genome-wide optical pooled screen. (C) Correlation between the PHATE potential distance from the clustering using the fine-tuned model and the adjusted FDR p-value from the Kotliar study, noting genes whose expression significantly increased or decreased along with infection. (D) Correlation between the PHATE potential distance from the supervised clustering and the number of mass spectrometry studies that identified the genes as an interactor with an Ebola virus protein. (E) Venn diagram showing overlap between top optical pooled screen hits, genes that were present in at least one mass spectrometry study, and differentially expressed genes from Kotliar et al's single-cell RNA sequencing study. (F) Correlation between the PHATE potential distance from the supervised clustering and the 95th percentile z-score for each gene in other virus genetic screens, see (G). (G) Unsupervised clustering of hits from genome-wide virus genetic screens, hierarchical clustering performed using cosine distance.

bioRxiv preprint doi: https://doi.org/10.1101/2024.04.06.588168; this version posted April 7, 2024. The copyright holder for this preprint (which was not certified by peer review) is the author/funder, who has granted bioRxiv a license to display the preprint in perpetuity. It is made available under aCC-BY 4.0 International license.

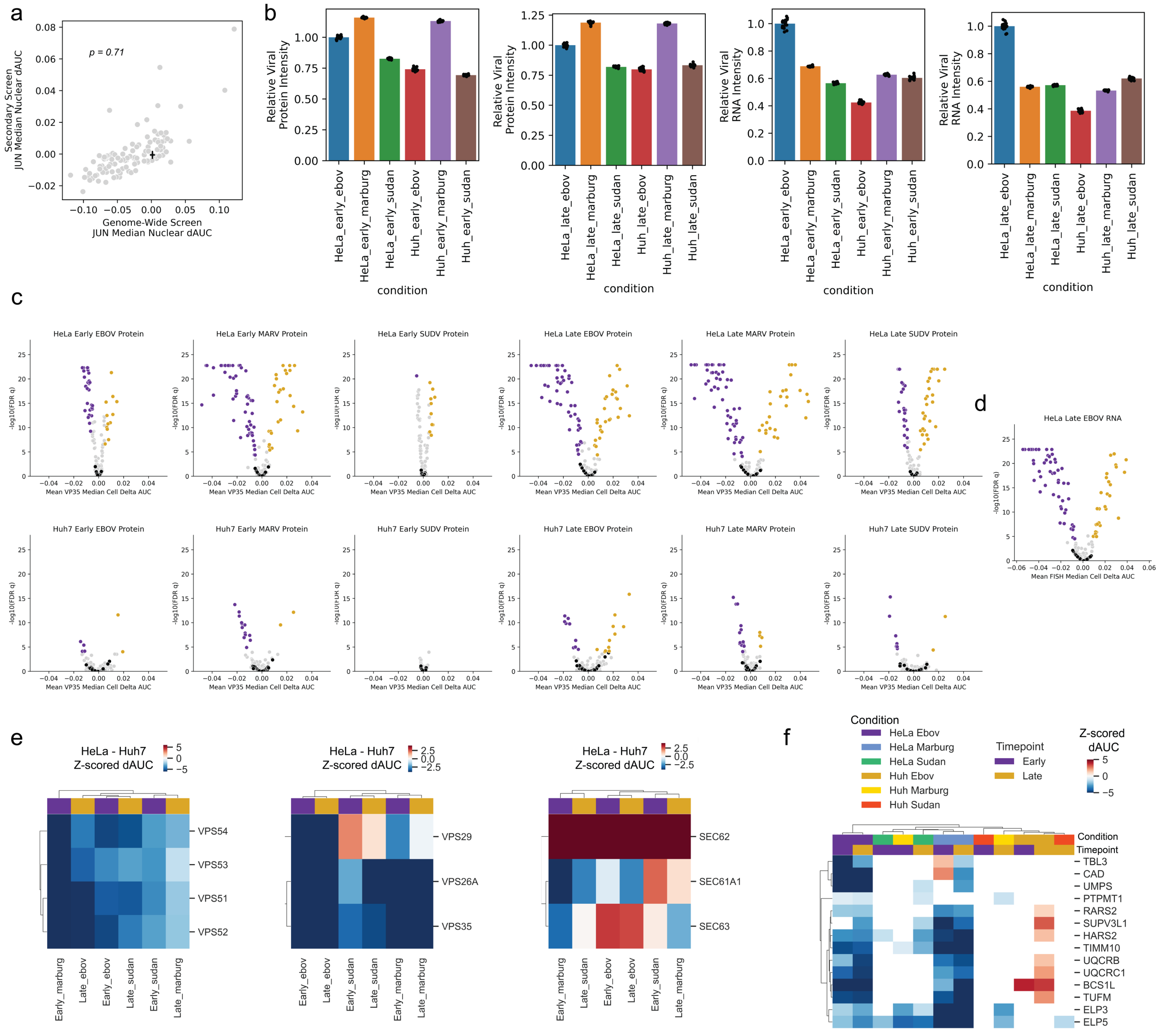

Figure S4. (A) Correlation between genome-wide c-Jun median nuclear delta AUC scores and secondary screen delta AUC scores; black lines indicate standard deviation for non-targeting control sgRNAs in each screen centered around the mean value for non-targeting sgRNAs in the screen. (B) Secondary screen mean viral protein (VP35 for EBOV and SUDV or VP40 for MARV) and RNA intensities in non-targeting control sgRNAs relative to HeLa cells infected with EBOV. (C) Volcano plots for VP35 (EBOV, SUDV) or VP40 (MARV) protein expression in each of the twelve screening conditions. (D) Volcano plot for viral VP35 RNA levels in HeLa cells at the late timepoint condition. (E) Heatmaps showing the difference between HeLa cell and Huh7 cell z-scored delta AUCs for members of the GARP, retromer, and the Sec61 complex. Hierarchical clustering performed using Euclidean distance. (F) Heatmap showing z-scored delta AUC values for genes identified as enriched for a punctate phenotype in the genome-wide screen and also included in secondary screens (white cells indicate conditions where p > 0.05). Hierarchical clustering performed using Pearson correlations.
